# Supplementary material for: Health information technology uses for primary prevention in preventive medicine: a scoping review protocol
Source: BMJ Open. 2018 Oct 4;8(9):e023428. doi: 10.1136/bmjopen-2018-023428 (PMC6173258; doi:10.1136/bmjopen-2018-023428)
Supplement: Author's manuscript [file bmjopen-2018-023428.draft_revisions.pdf]

BMJ Open

BMJ Open is committed to open peer review. As part of this commitment we make the peer review history of every article we publish publicly available.

When an article is published we post the peer reviewers' comments and the authors' responses online. We also post the versions of the paper that were used during peer review. These are the versions that the peer review comments apply to.

The versions of the paper that follow are the versions that were submitted during the peer review process. They are not the versions of record or the final published versions. They should not be cited or distributed as the published version of this manuscript.

BMJ Open is an open access journal and the full, final, typeset and author-corrected version of record of the manuscript is available on our site with no access controls, subscription charges or pay-per-view fees (http://bmjopen.bmj.com).

If you have any questions on BMJ Open's open peer review process please email info.bmjopen@bmj.com

BMJ Open

Health information technology uses for primary prevention in preventive medicine: A scoping review protocol

Journal:	*BMJ Open*
Manuscript ID	bmjopen-2018-023428
Article Type:	Protocol
Date Submitted by the Author:	05-Apr-2018

Complete List of Authors:	Alturkistani, Abrar; Imperial College London, Primary Care and Public Health<br>Majeed, Azeem; Imperial College, Primary Care<br>Car, Josip; Imperial College London, Primary Care and Social Medicine<br>Brindley, David; University of Oxford, Paediatrics; University of Oxford, Said Buisness School<br>Wells, Glenn; Oxford Academic Health Science Centre<br>Meinert, Edward; Imperial College London, Primary Care and Public Health; University of Oxford, Paediatrics

Keywords:	PREVENTIVE MEDICINE, Digital Health, Health Information Technologies, PUBLIC HEALTH

SCHOLARONE™
Manuscripts

For peer review only

Health information technology uses for primary prevention in preventive medicine: A scoping review protocol

Abrar Alturkistani, Azeem Majeed, Josip Car, David Brindley, Glenn Wells, Edward Meinert

Global eHealth Unit, Department of Public Health and Primary Care, School of Public Health, Imperial College London, W6 8RP London, United Kingdom Abrar Alturkistani Research Assistant Department of Public Health and Primary Care, School of Public Health, Imperial College London, W6 8RP London, United Kingdom Azeem Majeed Professor of Primary Care Global eHealth Unit, Department of Public Health and Primary Care, School of Public Health, Imperial College London, W6 8RP London, United Kingdom Josip Car Director of the Global eHealth Unit Department of Paediatrics, University of Oxford, OX3 9DU Oxford, United Kingdom David Brindley Senior Research Fellow Oxford Academic Health Science Centre, OX4 4GA Oxford, United Kingdom Glenn Wells Chief Operating Officer Department of Paediatrics, University of Oxford, OX3 9DU Oxford, United Kingdom Edward Meinert Sir David Cooksey Fellow in Healthcare Translation Global eHealth Unit, Department of Public Health and Primary Care, School of Public Health, Imperial College London, W6 8RP London, United Kingdom Edward Meinert Honorary Research Fellow

Correspondence to:
Edward Meinert e.meinert14@imperial.ac.uk edward.meinert@paediatrics.ox.ac.uk
00447824446808

Word Count: 2709

Keywords: Scoping Review, Health Information Technologies, Preventive Medicine, Digital Health

For peer review only

ABSTRACT

Introduction: The use of health information technologies (HITs) has been associated with positive benefits such as improved health outcomes and improved health services. Results from empirical studies reported potential benefits of HITs in preventive medicine measures such as primary prevention. This review will examine the broad range of HITs and their uses and effectiveness in primary prevention.

Methods and analysis: We will conduct searches in relevant databases (MEDLINE, EMBASE, the Cochrane Methodology Register and Cochrane Database of Systematic Reviews.) using Arksey and O'Malley's scoping review methodology. The scoping review will include all study designs to identify the literature on health information technology uses. Two reviewers will independently screen the literature following our screening criteria and using a data abstraction form. Findings will be summarized quantitatively (using numerical counts of HITs) and qualitatively (using narrative synthesis).

Ethics and dissemination
The study will synthesize data from published literature and will not require an ethical approval. The results of the review will be disseminated through a peer-reviewed journal.

Registration
The review protocol will be submitted to PROSPERO.

For peer review only

For peer review only - http://bmjopen.bmj.com/site/about/guidelines.xhtml

STRENGTHS AND LIMITATIONS OF THIS STUDY

- A strength of this study is that it will conduct a comprehensive review of the relevant databases to help inform healthcare professionals, researchers and policy makers about the latest uses of HITs for preventive medicine purposes.
- A strength of this study will also help identify gaps in the literature concerning HITs and their effectiveness and uses in preventive medicine.
- A limitation of this study is that it will only include English language publications.
- A limitation of this study is that it will not perform a formal quality assessment of included studies.

BACKGROUND

Health information technology (HIT) include technologies that enable health information to be stored, disseminated and analysed [1] and are increasingly used to improve the health of patients and populations. Popular examples of HITs include electronic health records, smartphone health applications (apps) and electronic prescriptions (E-prescribing) [1]. Evidence from existing systematic reviews and empirical studies found positive effects of using HITs in improving health outcomes. Research shows that HITs can not only improve health outcomes but also contribute to preventing disease and improving preventive medicine practices. Preventive medicine is the practice that focuses on keeping individuals healthy and its goal is to "protect, promote, and maintain health and well-being and to prevent disease, disability, and death" [2]. Primary prevention is one of the preventive medicine measures and it is defined as the prevention of "the initial occurrence of a disorder" by the World Health Organization [3]. Despite the potential benefits HITs can have to improve primary prevention, and the availability of studies about the use of HITs for primary prevention, there are currently no studies that comprehensively review the different types of HITs and their uses in primary prevention.

HITs have seen a growing interest in the literature in recent years and have been repeatedly associated with preventing disease [4-6] improving health outcomes [7] improving data collection, and the potential to substantially advance healthcare research [8-10]. As different HITs proliferate, questions about their effectiveness are being raised. HITs are associated with positive outcomes in healthcare in general such as "efficiency of care", "effectiveness of care" and "patient safety" [10].

Reviews related to the use of HITs in primary prevention focus on only one or two types of health information technologies (e.g. telephone-based interventions only) [11]. Most of the studies that focus on primary prevention outcomes focus on one tool or method of HITs like electronic health records [8] or mobile health technologies [5]. However, these studies are not representative of the whole range of HITs that can be used in primary prevention. In addition, some of the currently available reviews, even if includes more than one HIT, only focuses on one or two primary prevention outcomes (e.g. smoking) [9].

This review will focus on gathering information on what is available rather than which interventions work best. This general focus allows the examination of all the available interventions in health information technologies. In this review, we will map out the findings and results of studies published about health information technologies and their uses in primary prevention preventive

For peer review only - http://bmjopen.bmj.com/site/about/guidelines.xhtml

medicine. A scoping review can help clarify to what extent are HITs used for primary prevention purposes and what is the range of the HITs available. We will synthesize the available evidence to inform how technology could be developed to impact primary prevention in preventive medicine. In this protocol we have reviewed some HITs used for primary prevention in Table 1, as example of the scoping review outcomes that will result from the study.

**Intervention**	**Primary prevention uses**	**Description of intervention**
Mobile phone messaging (SMS or MMS)	Smoking cessation (Rodgers et al., 2005)	Personalized smoking-related and general healthy behaviour-related messages sent to participants as part of a smoking cessation programme. The intervention had other features like being able to text other participants, requesting texts on quitting-related tips and taking polls and quizzes about smoking [9].
	Adherence in taking vitamin C for preventive reasons (Cocosila et al., 2009)	Text message sent from a virtual character to remind to take a Vitamin C pill to participants, where they are expected to "acknowledge" the reminder. If the text was acknowledged an encouraging message is sent, if not, a reminder message in sent. The encouraging messages were described as amusing while the reminder messages were described as "non-amusing" [12].
	Healthy behaviour in children (Shapiro et al., 2008)	Feedback text messages sent as part of a programme to promote healthy behaviors in children (to increase physical activity, reduce sugary beverage consumption and screen time). The feedback text messages were sent once the participants send a text message informing their achievement of predetermined healthy behaviour related goals [13].
Internet-based interventions	Smoking prevention (Buller et al., 2008)	Internet-based programme for school-children that uses "audio narration, graphics, animation, sound effects, and music" to deliver lessons for smoking prevention with survey questions asked to personalize the lessons for the student [14].
	HIV prevention (Kasatpibal et al., 2014)	Internet-based educational programme that uses "texts, pictures, animation, animated cartoons, videos, message boards, and exercise" to teach about the risks of HIV for men who have sex with men [15].

For peer review only - http://bmjopen.bmj.com/site/about/guidelines.xhtml

	Obesity prevention (Rerksuppaphol and Rerksuppaphol, 2017)	Internet-based programme for school-aged children to track weight and nutrition-related information and provide personalized information about nutrition and physical activity based on the user's weight/health status [16].
Telephone-based intervention	Postpartum depression prevention (Lewis et al., 2012)	A telephone-based intervention to increase exercise (known to prevent postpartum depression) as part of a prevention programme. The telephone-based intervention is used to inform and educate the participants about exercising, explain exercise recommendations, and encourage participants to maintain exercising [17].
Smartphone application (app)	Diabetes prevention (Fukuoka et al., 2015)	An interactive app with a "self-monitoring" tool and a list of tasks for activities that can prevent diabetes like physical activity. The app also provides encouraging feedback based on the user's input [18].

Table 1: Description of preliminary list of existing health information technology uses in primary prevention

AIMS & OBJECTIVES

The aim of this review is to provide an overview of all HITs that are used for the purpose of primary prevention or to achieve primary prevention outcomes. Through this review, the available HITs, their uses, limitations and gaps in the literature regarding their use in primary prevention will be reported. The objectives of the review are the following:

- To identify the health information technologies that are used for primary prevention.
- To identify the primary prevention patient outcomes that are improved by the use of health information technologies.
- Map out the ways health information technologies are changing/improving primary prevention compared to standard/traditional methods.

METHODS

To outline the protocol of the forthcoming scoping review, we will be using the Preferred Reporting Items for Systematic Reviews and Meta-analysis for Protocols (PRISMA-P) (Appendix 1).

Protocol Design

We will use the Arksey and O'Malley methodological framework for scoping reviews in performing the review. The framework recommends following six steps to conduct a scoping review: (1) identifying the research question; (2) identifying relevant studies; (3) selecting studies; (4) charting the data; and (5) collating, summarising and reporting the results [19]. This framework, although relatively new (2003), is the first methodological framework for scoping reviews and it has been widely used for this purpose.

For peer review only

For peer review only - http://bmjopen.bmj.com/site/about/guidelines.xhtml

Stage 1: Identifying the research question

The preliminary research (Table 1) revealed that there are no review studies that reviewed the different HIT approaches used in primary prevention and exposed a research gap that motivated the focus of this protocol. The main research question and the secondary research questions of the scoping review are displayed in Table 2.

Primary Research Questions	Secondary Research Questions
What health information technologies are used in primary prevention preventive medicine to improve individuals/patients health outcomes?	• What tools and innovations of health information technologies are used in primary prevention preventive medicine?<br>• What primary prevention preventive medicine patient/individual health outcomes are improved by the use of HITs?<br>• How are the use of HITs changing/improving primary prevention preventive medicine compared to standard/traditional methods?

Table 2: Scoping review primary and secondary research questions

Stage 2: Identifying relevant studies

Search strategy

For the scoping review, we will conduct searches in relevant electronic databases: MEDLINE, EMBASE, the Cochrane Methodology Register and Cochrane Database of Systematic Reviews. The literature search strategy used for Medline can be found in (Appendix 2), including the medical subheadings (MeSH) and free text terms used to perform the search. The search strategy will be modified for each database and it will not be limited in terms of year or study design. Only studies in English language will be reviewed. Apart from electronic databases, we will also search reference lists of the studies selected for full text reading to supplement the search.

Stage 3: Study Selection

Screening of the studies will be performed by two suitably experienced/qualified reviewers and in two levels. Table 3 outlines the inclusion criteria that will be used by the reviewers to determine the studies that will be included. The citation management software program; EndNote X8.2 (Clarivate Analytics, USA), will be used to manage records and data and to remove duplicates. The first screening will involve screening the title and abstracts. Using two reviewers will ensure that all relevant articles are included. The reviewers will use the pre-defined relevance criteria to determine relevant studies. In the second round of screening, the reviewers will perform full text reading of the studies identified in the previous round. Conflicts and discrepancies will be resolved by discussing with a third party.

For peer review only - http://bmjopen.bmj.com/site/about/guidelines.xhtml

Inclusion Criteria	
**Population**	• Users of the health information technologies will include individuals or patients who are treated with primary prevention preventive medicine.
**Intervention**	• All health information technologies (e.g. electronic health records, telemedicine, text messages, computerized decision support systems...).
**Comparator**	• Studies using non-health information technology interventions<br>• Studies using traditional or usual method as a comparator to health information technology<br>• Studies without a comparator
**Outcomes**	• Any primary prevention outcome that prevents a disease or a health-threatening condition or a behaviour before it occurs (e.g. chronic disease prevention, smoking prevention, obesity prevention)
**Study Type**	• Any study type; experimental (randomised controlled trials (RCTs), quasi-RCTs, non-RCTs), quasi-experimental (controlled before after, interrupted time series) and observational (cohort, case control, cross-sectional) and review (systematic review, meta-analysis scoping review) studies.<br>• Only published literature will be included in the review.<br>• Only publications in English will be included.<br>• There will be no restrictions to calendar date.

Table 3: Review inclusion criteria

Exclusion Criteria

- Interventions that focus on secondary or tertiary prevention will be excluded to keep the focus on the primary prevention interventions only.
- Publications that are not in English will be excluded.

Stage 4: Charting the data

For peer review only - http://bmjopen.bmj.com/site/about/guidelines.xhtml

Two reviewers will independently extract the data and vigilantly review the studies based on the data abstraction form (Appendix 3). We assume that studies identified for this review will include basic study information like: first author and year of publication and will include information about the health information technology intervention and the methods used in the study. Quality assessment of the studies will not be completed since it is not one of the activities performed in a scoping review [19].

Stage 5: Collating, summarizing and reporting the results

The studies identified from this scoping review will be summarized and analysed using quantitative and qualitative methods. In terms of quantitative methods, we will report simple numerical counts of information such as: the total number of studies, types of primary prevention HIT interventions, descriptions of the study samples and regarding qualitative methods, we will conduct a narrative synthesis to provide an overview of the breadth of the literature and to identify gaps that may need further research. To address the three research questions of the review, we will analyse the data following three synthesis objectives: to identify the health information technologies that are used for primary prevention, to identify the primary prevention patient outcomes that are improved by the use of health information technologies and to map out the ways health information technologies are changing/improving primary prevention compared to standard/traditional methods. Table 3 displays each of the synthesis objectives of the review followed by the method, guide questions and outputs that will be used to achieve them.

**Synthesis objective**	**Method**	**Guide Questions**	**Outputs**
**1. To identify the health information technologies that are used for primary prevention.**	We will summarize the identified studies by the health information technology used	What is the health information technology?<br>What is the purpose of the health information technology and how does the purpose contribute to primary prevention?<br>In what setting is the primary prevention technology used? (e.g. healthcare, community setting…etc)<br>What type of evidence does the	A list of the health information technologies used for primary prevention purposes.<br>A list of the settings that the health information technologies are used in categorization of the primary prevention related outcomes.

For peer review only - http://bmjopen.bmj.com/site/about/guidelines.xhtml

		study provide for primary prevention related health outcomes?	
**2. To identify the primary prevention patient outcomes that are improved by the use of health information technologies.**	We will strictly identify the studies that reported significant improved patient outcomes as a result of using health information technologies	What are the studies that reported significant improved patient outcomes and what is the criteria they used to represent significance?<br>How health information technologies that improve patient outcomes are used to improve primary prevention measures?<br>Are there any disadvantages of using the health information technologies for primary prevention?<br>Can the health information technology be translated and used in different healthcare-related settings?	Identification of the health information technologies that contribute significant improved patient outcomes in the literature. A thematic report of the health information technology uses in primary prevention.

For peer review only

**3. Map out the ways health information technologies are changing/improving primary prevention compared to standard/traditional methods.**	We will identify the articles that compare health information technology interventions to traditional or standard interventions	Did the study compare primary prevention health outcomes to other standard or traditional methods of primary prevention?<br>What outcomes did the study report to compare the health information technologies to other methods?<br>How long were the health information technologies and other methods compared for?	A summary of the health information technologies that were reported to have superior primary prevention outcomes when compared to traditional or standard methods to map out the specific health information technologies that have been compared to traditional or standard methods of primary prevention.

Table 4: Data analysis plan by the synthesis objectives and anticipated outputs

For peer review only

Patient and Public Involvement

Research interests identified and prioritised by the members of the public in a workshop by the European Scientific Institute, on July 2018 were used to guide specifications of this research.

Ethics and dissemination

The proposed scoping review has the potential to improve research and inform policy makers, healthcare providers, clinicians and researchers on how health information technologies are used in preventive medicine. This scoping review could help advance research by showing the type of evidence and strategies available and by highlighting the need for further research in the field. This scoping review will provide a platform to list out the different health information technologies studied in the literature for their uses in primary prevention.

Due to the use of the publicly available, published data, this study will not require an ethical approval.

Authors' contributions

AA and EM participated in the design and development of the protocol. AA and EM drafted the manuscript. AM, JC, DB and GW reviewed the second draft. AA and EM incorporated and addressed the feedback from the authors. All authors read and approved the final manuscript.

Acknowledgements

We thank the medical librarians at Imperial College, Charing Cross campus for advising on search strategies and available resources.

Competing interests

All authors completed the ICMJE uniform disclosure form at www.icmje.org/coi_disclosure.pdf. Financial support was obtained from the Sir David Cooksey Fellowship in Healthcare Translation at the University of Oxford. There are no relevant conflicts of interest, financial or other types of relationships that may influence the manuscript declared by authors. Authors do not have any patents and are not associated to any conditions or circumstances that may lead to conflicts of interest.

Funding statement

The study was funded by the Sir David Cooksey Fellowship in Healthcare Translation at the University of Oxford.

For peer review only

References

1. HealthIT.gov. Basics of Health IT. https://www.healthit.gov/patients-families/basics-health-it Accessed Feb 6 2018.
2. American College of Preventive Medicine. Preventive Medicine [Internet]. acpm.org. [cited 2018 Mar 27]. Available from: http://www.acpm.org/page/preventivemedicine
3. Kickbusch I, Nutbeam D. Health promotion glossary. World Health Organization; 1998.
4. Vodopivec-Jamsek V, de Jongh T, Gurol-Urganci I, Atun R, Car J. Mobile phone messaging for preventive health care. Cochrane Database Syst Rev. 2012;doi:10.1002/14651858.CD007457.pub2
5. Gurol-Urganci I, de Jongh T, Vodopivec-Jamsek V, Car J, Atun R. Mobile phone messaging for communicating results of medical investigations. Cochrane Database Syst Rev. 2012;doi:10.1002/14651858.CD007456.pub2
6. Whittaker R, McRobbie H, Bullen C, Rodgers A, Gu Y. Mobile phone-based interventions for smoking cessation. Cochrane Database Syst Rev. 2016; doi:10.1002/14651858.CD006611.pub4
7. Chaudhry B, Wang J, Wu S, Maglione M, Mojica W, Roth E, et al. Systematic review: impact of health information technology on quality, efficiency, and costs of medical care. Ann Intern Med. 2006;144(10):742–52.
8. Miriovsky BJ, Shulman LN, Abernethy AP. Importance of health information technology, electronic health records, and continuously aggregating data to comparative effectiveness research and learning health care. J Clin Oncol Off J Am Soc Clin Oncol. 2012;doi:10.1200/JCO.2012.42.8011
9. Rodgers A, Corbett T, Bramley D, Riddell T, Wills M, Lin R-B, et al. Do u smoke after txt? Results of a randomised trial of smoking cessation using mobile phone text messaging. Tob Control. 2005;doi: 10.1136/tc.2005.011577
10. Buntin MB, Burke MF, Hoaglin MC, Blumenthal D. The Benefits Of Health Information Technology: A Review Of The Recent Literature Shows Predominantly Positive Results. Health Aff (Millwood). 2011;doi:10.1377/hlthaff.2011.0178
11. Posadzki P, Mastellos N, Ryan R, Gunn LH, Felix LM, Pappas Y, et al. Automated telephone communication systems for preventive healthcare and management of long-term conditions. Cochrane Database Syst Rev. 2016;doi:10.1002/14651858.CD009921.pub2
12. Cocosila M, Archer N, Haynes RB, Yuan Y. Can wireless text messaging improve adherence to preventive activities? Results of a randomised controlled trial. Int J Med Inf. 2009;doi:10.1016/j.ijmedinf.2008.07.011

For peer review only - http://bmjopen.bmj.com/site/about/guidelines.xhtml

13. Shapiro JR, Bauer S, Hamer RM, Kordy H, Ward D, Bulik CM. Use of text messaging for monitoring sugar-sweetened beverages, physical activity, and screen time in children: a pilot study. J Nutr Educ Behav. 2008;doi:10.1016/j.jneb.2007.09.014
14. Buller DB, Borland R, Woodall WG, Hall JR, Hines JM, Burris-Woodall P, et al. Randomized trials on consider this, a tailored, internet-delivered smoking prevention program for adolescents. Health Educ Behav Off Publ Soc Public Health Educ. 2008;doi:10.1177/1090198106288982
15. Kasatpibal N, Viseskul N, Srikantha W, Fongkaew W, Surapagdee N, Grimes RM. Effects of Internet-based instruction on HIV-prevention knowledge and practices among men who have sex with men. Nurs Health Sci. 2014;doi:10.1111/nhs.12135
16. Rerksuppaphol L, Rerksuppaphol S. Internet Based Obesity Prevention Program for Thai School Children- A Randomized Control Trial. J Clin Diagn Res JCDR. 2017;doi:10.7860/JCDR/2017/21423.9368
17. Lewis BA, Gjerdingen DK, Avery MD, Guo H, Sirard JR, Bonikowske AR, et al. Examination of a telephone-based exercise intervention for the prevention of postpartum depression: design, methodology, and baseline data from The Healthy Mom study. Contemp Clin Trials. 2012;doi:10.1016/j.cct.2012.07.015
18. Fukuoka Y, Gay CL, Joiner KL, Vittinghoff E. A Novel Diabetes Prevention Intervention Using a Mobile App: A Randomized Controlled Trial With Overweight Adults at Risk. Am J Prev Med. 2015;doi:10.1016/j.amepre.2015.01.003
19. Arksey H, O'Malley L. Scoping studies: towards a methodological framework. Int J Soc Res Methodol. 2005;doi:10.1080/1364557032000119616

For peer review only

Appendices:

Appendix 1: Table displaying the PRISMA-P 2015 Checklist

PRISMA-P 2015 Checklist

This checklist to be used for the Systematic Reviews protocol submission was adapted from Table 3 in Moher D et al: Preferred reporting items for systematic review and meta-analysis protocols (PRISMA-P) 2015 statement. Systematic Reviews 2015 4:1

Section and Topic	#	Checklist Item	Information reported		Page number(s)
			Yes	No	
**Administrative information**					
**Title**					
identification	1a	Identify the report as a protocol of a systematic review	☐	☒	
Update	1b	If the protocol is for an update of a previous systematic review, identify as such	☐	☒	
**Registration**	2	If registered, provide the name of the registry (such as PROSPERO) and registration number	☐	☒	
**Authors**					
Contact	3a	Provide name, institutional affiliation, e-mail address of all protocol authors; provide physical mailing address of corresponding author	☒	☐	1
Contributions	3b	Describe contributions of protocol authors and identify the guarantor of the review	☒	☐	11
**Amendments**	4	If the protocol represents an amendment of a previously completed or published protocol, identify as such and list changes; otherwise, state plan for documenting important protocol amendments	☐	☒	
**Support**					
Sources	5a	Indicate sources of financial or other support for the review	☒	☐	11

For peer review only

Section and Topic	#	Checklist Item	Information reported		Page number(s)
			Yes	No	
Sponsor	5b	Provide name for the review funder and/or sponsor	☒	☐	11
Role of Sponsor or Funder	5c	Describe roles of funder(s), sponsor(s), and/or institution(s), if any, in developing the protocol	☒	☐	11
**Introduction**					
Rationale	6	Describe the rationale for the review in the context of what is already known	☒	☐	3-4
Objectives	7	Provide an explicit statement of the question(s) the review will address with reference to participants, interventions, comparators, and outcomes (PICO)	☒	☐	6
**Methods**					
Eligibility criteria	8	Specify the study characteristics (such as PICO, study design, setting, time frame) and report characteristics (such as years considered, language, publication status) to be used as criteria for eligibility for the review	☒	☐	7-8
Information sources	9	Describe all intended information sources (such as electronic databases, contact with study authors, trial registers or other grey literature sources) with planned dates of coverage	☒	☐	6-7
Search strategy	10	Present draft of search strategy to be used for at least one electronic database, including planned limits, such that it could be repeated	☒	☐	17
Study records:					
Data management	11a	Describe the mechanism(s) that will be used to manage records and data throughout the review	☒	☐	7
Selection process	11b	State the process that will be used for selecting studies (such as two independent reviewers) through each phase of the review (that is, screening, eligibility and inclusion in meta-analysis)	☒	☐	7

Section and Topic	#	Checklist Item	Information	Page

For peer review only - http://bmjopen.bmj.com/site/about/guidelines.xhtml

			reported		number(s)
			Yes	No	
Data collection process	11c	Describe planned method of extracting data from reports (such as piloting forms, done independently, in duplicate), any processes for obtaining and confirming data from investigators	☒	☐	7, 18
**Data items**	12	List and define all variables for which data will be sought (such as PICO items, funding sources), any pre-planned data assumptions and simplifications	☒	☐	7-8
**Outcomes and prioritization**	13	List and define all outcomes for which data will be sought, including prioritization of main and additional outcomes, with rationale	☒	☐	7-10 18
**Risk of bias in individual studies**	14	Describe anticipated methods for assessing risk of bias of individual studies, including whether this will be done at the outcome or study level, or both; state how this information will be used in data synthesis	☐	☒	
**Data synthesis**	15a	Describe criteria under which study data will be quantitatively synthesised	☐	☒	
	15b	If data are appropriate for quantitative synthesis, describe planned summary measures, methods of handling data and methods of combining data from studies, including any planned exploration of consistency (such as I2 , Kendall's τ)	☐	☒	
	15c	Describe any proposed additional analyses (such as sensitivity or subgroup analyses, meta-regression)	☐	☒	
	15d	If quantitative synthesis is not appropriate, describe the type of summary planned	☐	☒	
**Meta bias(es)**	16	Specify any planned assessment of meta-bias(es) (such as publication bias across studies, selective reporting within studies)	☐	☒	
**Confidence in cumulative evidence**	17	Describe how the strength of the body of evidence will be assessed (such as GRADE)	☐	☒	

For peer review only

Appendix 2: Proposed MEDLINE Literature Search Strategy

Concept	Medical Subject Headings (MeSH)	Search terms
**Health Information Technologies**	Medical Informatics/	electronic patient record* OR electronic medical record* OR personal health record* OR Health information exchange or technology OR telemedicine OR text message* OR sms OR telephone OR computerized decision support system OR public health informatic* OR cellular phone* OR smartphone* OR mobile* OR ipad* or computer-assisted OR user-computer interface OR personal digital assistant OR computer* OR handheld OR electronic wearable device* OR electronic wearable technology OR data
**Primary Prevention**	Quality of Life/<br>tobacco use/<br>smoking/<br>dietary services/<br>preventive health services/ early intervention (education)/<br>early medical intervention/<br>health education/<br>primary prevention/<br>immunization/	exercise OR physical activity OR diet OR healthy behavior* OR weightloss OR weight change OR weight reduction OR weight management OR weight gain OR smoking cessation OR disease prevention

For peer review only

Appendix 3: Data abstraction form

<table>
<tr><td colspan="3">Reviewer</td><td>Date</td></tr>
<tr><td colspan="4">Scoping review of Health information technology used for primary prevention in preventive medicine</td></tr>
<tr><td colspan="4">Publication Information</td></tr>
<tr><td colspan="3">Study</td><td>First Author</td></tr>
<tr><td colspan="2">Year of Publication</td><td colspan="2">Journal</td></tr>
<tr><td colspan="2">Country</td><td colspan="2">Discipline</td></tr>
<tr><td>Health information technology(ies) studied</td><td colspan="3"></td></tr>
<tr><td rowspan="2">Objective 1</td><td colspan="3">General description of the health information technology(ies) studied</td></tr>
<tr><td colspan="3">The primary prevention purpose of the health information technology</td></tr>
<tr><td>Objective 2</td><td colspan="3">Primary prevention patient outcome(s) studied</td></tr>
<tr><td>Objective 3</td><td colspan="3">Is there a comparator to the health information technology, if so, how is it different than the comparator?</td></tr>
</table>

For peer review only - http://bmjopen.bmj.com/site/about/guidelines.xhtml

BMJ Open

Health information technology uses for primary prevention in preventive medicine: A scoping review protocol

Journal:	*BMJ Open*
Manuscript ID	bmjopen-2018-023428.R1
Article Type:	Protocol
Date Submitted by the Author:	10-Jul-2018
Complete List of Authors:	Alturkistani, Abrar; Imperial College London, Primary Care and Public Health<br>Majeed, Azeem; Imperial College, Primary Care<br>Car, Josip; Imperial College London, Primary Care and Social Medicine<br>Brindley, David; University of Oxford, Paediatrics; University of Oxford, Said Buisness School<br>Wells, Glenn; Oxford Academic Health Science Centre<br>Meinert, Edward; Imperial College London, Primary Care and Public Health; University of Oxford, Paediatrics
**Primary Subject Heading**:	General practice / Family practice
Secondary Subject Heading:	Diagnostics
Keywords:	PREVENTIVE MEDICINE, Digital Health, Health Information Technologies, PUBLIC HEALTH

SCHOLARONE™
Manuscripts

Health information technology uses for primary prevention in preventive medicine: A scoping review protocol

Abrar Alturkistani, Azeem Majeed, Josip Car, David Brindley, Glenn Wells, Edward Meinert

Global eHealth Unit, Department of Public Health and Primary Care, School of Public Health, Imperial College London, W6 8RP London, United Kingdom Abrar Alturkistani Research Assistant Department of Public Health and Primary Care, School of Public Health, Imperial College London, W6 8RP London, United Kingdom Azeem Majeed Professor of Primary Care Global eHealth Unit, Department of Public Health and Primary Care, School of Public Health, Imperial College London, W6 8RP London, United Kingdom Josip Car Director of the Global eHealth Unit Department of Paediatrics, University of Oxford, OX3 9DU Oxford, United Kingdom David Brindley Senior Research Fellow Oxford Academic Health Science Centre, OX4 4GA Oxford, United Kingdom Glenn Wells Chief Operating Officer Department of Paediatrics, University of Oxford, OX3 9DU Oxford, United Kingdom Edward Meinert Sir David Cooksey Fellow in Healthcare Translation Global eHealth Unit, Department of Public Health and Primary Care, School of Public Health, Imperial College London, W6 8RP London, United Kingdom Edward Meinert Honorary Research Fellow

Correspondence to:
Edward Meinert e.meinert14@imperial.ac.uk edward.meinert@paediatrics.ox.ac.uk
00447824446808

Word Count: 2800

Keywords: Scoping Review, Health Information Technologies, Preventive Medicine, Digital Health

For peer review only

ABSTRACT

Introduction: The use of health information technologies (HITs) has been associated with positive benefits such as improved health outcomes and improved health services. Results from empirical studies reported potential benefits of HITs in preventive medicine measures such as primary prevention. This review will examine the broad range of HITs and their uses and effectiveness in primary prevention.

Methods and analysis: We will conduct searches in relevant databases (MEDLINE, EMBASE, the Cochrane Methodology Register, Cochrane Database of Systematic Reviews, CINAHL, SCOPUS and Web of Science) using Arksey and O'Malley's scoping review methodology. The scoping review will include all study designs to identify the literature on health information technology uses. Two reviewers will independently screen the literature following our screening criteria and using a data abstraction form. Findings will be summarized quantitatively (using numerical counts of HITs) and qualitatively (using narrative synthesis).

Ethics and dissemination

The study will synthesize data from published literature and will not require an ethical approval. The results of the review will be disseminated through a peer-reviewed journal.

Registration

Because the review method uses a scoping protocol, it is ineligible for submission to PROSPERO.

For peer review only

For peer review only - http://bmjopen.bmj.com/site/about/guidelines.xhtml

STRENGTHS AND LIMITATIONS OF THIS STUDY

- A strength of this study is that it will conduct a comprehensive review of the relevant databases to help inform healthcare professionals, researchers and policy makers about the latest uses of HITs for preventive medicine purposes.
- A strength of this study will also help identify gaps in the literature concerning HITs and their effectiveness and uses in preventive medicine.
- A limitation of this study is that it will only include English language publications.
- A limitation of this study is that it will not perform a formal quality assessment of included studies.

BACKGROUND

Health information technology (HIT) include technologies that enable health information to be stored, disseminated and analysed [1] and are increasingly used to improve the health of patients and populations. Popular examples of HITs include electronic health records, smartphone health applications (apps) and electronic prescriptions (E-prescribing) [1]. Evidence from existing systematic reviews and empirical studies found positive effects of using HITs in improving health outcomes. Research shows that HITs can not only improve health outcomes but also contribute to preventing disease and improving preventive medicine practices. Preventive medicine is the practice that focuses on keeping individuals healthy and its goal is to "protect, promote, and maintain health and well-being and to prevent disease, disability, and death" [2]. Primary prevention is one of the preventive medicine measures and it is defined as the prevention of "the initial occurrence of a disorder" by the World Health Organization [3]. Despite the potential benefits HITs can have to improve primary prevention, and the availability of studies about the use of HITs for primary prevention, there are currently no studies that comprehensively review the different types of HITs and their uses in primary prevention.

HITs have seen a growing interest in the literature in recent years and have been repeatedly associated with preventing disease [4-6] improving health outcomes [7] improving data collection, and the potential to substantially advance healthcare research [8-10]. As different HITs proliferate, questions about their effectiveness are being raised. HITs are associated with positive outcomes in healthcare in general such as "efficiency of care", "effectiveness of care" and "patient safety" [10].

Reviews related to the use of HITs in primary prevention focus on only one or two types of health information technologies (e.g. telephone-based interventions only) [11]. Most of the studies that focus on primary prevention outcomes focus on one tool or method of HITs like electronic health records [8] or mobile health technologies [5]. However, these studies are not representative of the whole range of HITs that can be used in primary prevention. In addition, some of the currently available reviews, even if includes more than one HIT, only focuses on one or two primary prevention outcomes (e.g. smoking) [9].

This review will focus on gathering information on what is available rather than which interventions work best. This general focus allows the examination of all the available interventions in health information technologies. In this review, we will map out the findings and results of studies published about health information technologies and their uses in primary prevention preventive

For peer review only - http://bmjopen.bmj.com/site/about/guidelines.xhtml

medicine. A scoping review can help clarify to what extent are HITs used for primary prevention purposes and what is the range of the HITs available. We will synthesize the available evidence to inform how technology could be developed to impact primary prevention in preventive medicine. In this protocol we have reviewed some HITs used for primary prevention in Table 1, as example of the scoping review outcomes that will result from the study.

**Intervention**	**Primary prevention uses**	**Description of intervention**
Mobile phone messaging (SMS or MMS)	Smoking cessation (Rodgers et al., 2005)	Personalized smoking-related and general healthy behaviour-related messages sent to participants as part of a smoking cessation programme. The intervention had other features like being able to text other participants, requesting texts on quitting-related tips and taking polls and quizzes about smoking [9].
	Adherence in taking vitamin C for preventive reasons (Cocosila et al., 2009)	Text message sent from a virtual character to remind to take a Vitamin C pill to participants, where they are expected to “acknowledge” the reminder. If the text was acknowledged an encouraging message is sent, if not, a reminder message in sent. The encouraging messages were described as amusing while the reminder messages were described as “non-amusing” [12].
	Healthy behaviour in children (Shapiro et al., 2008)	Feedback text messages sent as part of a programme to promote healthy behaviors in children (to increase physical activity, reduce sugary beverage consumption and screen time). The feedback text messages were sent once the participants send a text message informing their achievement of predetermined healthy behaviour related goals [13].
Internet-based interventions	Smoking prevention (Buller et al., 2008)	Internet-based programme for school-children that uses “audio narration, graphics, animation, sound effects, and music” to deliver lessons for smoking prevention with survey questions asked to personalize the lessons for the student [14].
	HIV prevention (Kasatpibal et al., 2014)	Internet-based educational programme that uses “texts, pictures, animation, animated cartoons, videos, message boards, and exercise” to teach about the risks of HIV for men who have sex with men [15].

For peer review only - http://bmjopen.bmj.com/site/about/guidelines.xhtml

	Obesity prevention (Rerksuppaphol and Rerksuppapphol, 2017)	Internet-based programme for school-aged children to track weight and nutrition-related information and provide personalized information about nutrition and physical activity based on the user's weight/health status [16].
Telephone-based intervention	Postpartum depression prevention (Lewis et al., 2012)	A telephone-based intervention to increase exercise (known to prevent postpartum depression) as part of a prevention programme. The telephone-based intervention is used to inform and educate the participants about exercising, explain exercise recommendations, and encourage participants to maintain exercising [17].
Smartphone application (app)	Diabetes prevention (Fukuoka et al., 2015)	An interactive app with a "self-monitoring" tool and a list of tasks for activities that can prevent diabetes like physical activity. The app also provides encouraging feedback based on the user's input [18].

Table 1: Description of preliminary list of existing health information technology uses in primary prevention

AIMS & OBJECTIVES

The aim of this review is to provide an overview of all HITs that are used for the purpose of primary prevention or to achieve primary prevention outcomes. Through this review, the available HITs, their uses, limitations and gaps in the literature regarding their use in primary prevention will be reported. The objectives of the review are the following:

- To identify the health information technologies that are used for primary prevention and to analyse both the benefits and risks achieved by their use.
- To identify the primary prevention patient outcomes that are impacted by the use of health information technologies.

METHODS

To outline the protocol of the forthcoming scoping review, we will be using the Preferred Reporting Items for Systematic Reviews and Meta-analysis for Protocols (PRISMA-P) (Appendix 1).

Protocol Design

We will use the Arksey and O'Malley methodological framework for scoping reviews in performing the review. The framework recommends following six steps to conduct a scoping review: (1) identifying the research question; (2) identifying relevant studies; (3) selecting studies; (4) charting the data; and (5) collating, summarising and reporting the results [19]. This framework is being used for this review because it applies a rapid form of knowledge synthesis, with the intent to identify the merits of the underlying research question. This form of review is intended to be a precursor for potential further work, as on initial analysis it is unclear if a more sophisticated review method is warranted.

For peer review only - http://bmjopen.bmj.com/site/about/guidelines.xhtml

Stage 1: Identifying the research question

The preliminary research (Table 1) revealed that there are no review studies that reviewed the different HIT approaches used in primary prevention and exposed a research gap that motivated the focus of this protocol. The main research question and the secondary research questions of the scoping review are displayed in Table 2.

Primary Research Questions	Secondary Research Questions
What health information technologies are used in primary prevention preventive medicine to impact individuals/patients health outcomes?	• What tools and innovations of health information technologies are used in primary prevention preventive medicine?<br>• What primary prevention preventive medicine patient/individual health outcomes are impacted by the use of HITs?<br>• What are the risks and benefits associated with HITs?<br>• How are the use of HITs changing/improving primary prevention preventive medicine compared to standard/traditional methods?

Table 2: Scoping review primary and secondary research questions

Stage 2: Identifying relevant studies

Search strategy

For the scoping review, we will conduct searches in relevant electronic databases: MEDLINE, EMBASE, the Cochrane Methodology Register, Cochrane Database of Systematic Reviews, CINAHL, SCOPUS and Web of Science. The initial literature search strategy used for Medline can be found in (Appendix 2), including the medical subheadings (MeSH) and free text terms used to perform the search. The search strategy will be modified for each database and further iterated as we explore the research question with changes captured in the review process. Studies will not be limited in terms of year or study design. Only studies in English language will be reviewed. Apart from electronic databases, we will also search reference lists of the studies selected for full text reading to supplement the search.

Stage 3: Study Selection

Screening of the studies will be performed by two suitably experienced/qualified reviewers and in two levels. Table 3 outlines the inclusion criteria that will be used by the reviewers to determine the studies that will be included. The citation management software program; EndNote X8.2 (Clarivate Analytics, USA), will be used to manage records and data and to remove duplicates. The first screening will involve screening the title and abstracts. Using two reviewers will ensure that all

For peer review only - http://bmjopen.bmj.com/site/about/guidelines.xhtml

relevant articles are included. The reviewers will use the pre-defined relevance criteria to determine relevant studies. In the second round of screening, the reviewers will perform full text reading of the studies identified in the previous round. Conflicts and discrepancies will be resolved by discussing with a third party.

Inclusion Criteria	
**Population**	• Users of the health information technologies will include individuals or patients who are treated with primary prevention preventive medicine.
**Intervention**	• All health information technologies (e.g. electronic health records, telemedicine, text messages, computerized decision support systems...).
**Comparator**	• Studies using non-health information technology interventions<br>• Studies using traditional or usual method as a comparator to health information technology<br>• Studies without a comparator
**Outcomes**	• Any primary prevention outcome that prevents a disease or a health-threatening condition or a behaviour before it occurs (e.g. chronic disease prevention, smoking prevention, obesity prevention)
**Study Type**	• Any study type; experimental (randomised controlled trials (RCTs), quasi-RCTs, non-RCTs), quasi-experimental (controlled before after, interrupted time series) and observational (cohort, case control, cross-sectional) and review (systematic review, meta-analysis scoping review) studies.<br>• Only publications in English will be included.<br>• There will be no restrictions to calendar date; we intend to capture a broad survey of technologies developed and therefore are not restricting date range.

Table 3: Review inclusion criteria

Exclusion Criteria

For peer review only - http://bmjopen.bmj.com/site/about/guidelines.xhtml

- Interventions that focus on secondary or tertiary prevention will be excluded to keep the focus on the primary prevention interventions only.
- Publications that are not in English will be excluded.

Stage 4: Charting the data

Two reviewers will independently extract the data and vigilantly review the studies based on the data abstraction form (Appendix 3). We assume that studies identified for this review will include basic study information like: first author and year of publication and will include information about the health information technology intervention and the methods used in the study. Following review of the primary studies types to be included in the review, an appropriate quality assessment standard shall be used to assess the quality of the included papers.

Stage 5: Collating, summarizing and reporting the results

The studies identified from this scoping review will be summarized and analysed using quantitative and qualitative methods. In terms of quantitative methods, we will report simple numerical counts of information such as: the total number of studies, types of primary prevention HIT interventions, descriptions of the study samples and regarding qualitative methods, we will conduct a narrative synthesis to provide an overview of the breadth of the literature and to identify gaps that may need further research. To address the three research questions of the review, we will analyse the data following three synthesis objectives: to identify the health information technologies that are used for primary prevention, to identify the primary prevention patient outcomes that are improved by the use of health information technologies and to map out the ways health information technologies are changing/improving primary prevention compared to standard/traditional methods. Table 4 displays each of the synthesis objectives of the review followed by the method, guide questions and outputs that will be used to achieve them.

**Synthesis objective**	**Method**	**Guide Questions**	**Outputs**

For peer review only

**1. To identify the health information technologies that are used for primary prevention.**	We will summarize the identified studies by the health information technology used	What is the health information technology?<br>What is the purpose of the health information technology and how does the purpose contribute to primary prevention?<br>In what setting is the primary prevention technology used? (e.g. healthcare, community setting…etc)<br>What type of evidence does the study provide for primary prevention related health outcomes?	A list of the health information technologies used for primary prevention purposes.<br>A list of the settings that the health information technologies are used in categorization of the primary prevention related outcomes.
**2. To identify the primary prevention patient outcomes that are improved by the use of health information technologies.**	We will strictly identify the studies that reported significant improved patient outcomes as a result of using health information technologies	What are the studies that reported significant improved patient outcomes and what is the criteria they used to represent significance?<br>How health information technologies that improve patient outcomes are used to improve primary prevention measures?<br>Are there any disadvantages of using the health information technologies for	Identification of the health information technologies that contribute significant improved patient outcomes in the literature.<br>A thematic report of the health information technology uses in primary prevention.

For peer review only

		primary prevention? Can the health information technology be translated and used in different healthcare-related settings?	
**3. Map out the ways health information technologies are changing/improving primary prevention compared to standard/traditional methods.**	We will identify the articles that compare health information technology interventions to traditional or standard interventions	Did the study compare primary prevention health outcomes to other standard or traditional methods of primary prevention? What outcomes did the study report to compare the health information technologies to other methods? How long were the health information technologies and other methods compared for?	A summary of the health information technologies that were reported to have superior primary prevention outcomes when compared to traditional or standard methods to map out the specific health information technologies that have been compared to traditional or standard methods of primary prevention.

Table 4: Data analysis plan by the synthesis objectives and anticipated outputs

For peer review only

Patient and Public Involvement

Research interests identified and prioritised by the members of the public in a workshop by the European Scientific Institute, on July 2018 were used to guide specifications of this research.

Ethics and dissemination

The proposed scoping review has the potential to improve research and inform policy makers, healthcare providers, clinicians and researchers on how health information technologies are used in preventive medicine. This scoping review could help advance research by showing the type of evidence and strategies available and by highlighting the need for further research in the field. This scoping review will provide a platform to list out the different health information technologies studied in the literature for their uses in primary prevention.

Due to the use of the publicly available, published data, this study will not require an ethical approval.

Acknowledgements

We thank the medical librarians at Imperial College, Charing Cross campus for advising on search strategies and available resources. This work was supported by the Sir David Cooksey Fellowship in Healthcare Translation and the SENS Research Foundation.

Contributorship Statement

AA and EM participated in the design and development of the protocol. AA and EM drafted the manuscript. AM, JC, DB and GW reviewed the second draft. AA and EM incorporated and addressed the feedback from the authors. All authors read and approved the final manuscript. All authors completed the ICMJE uniform disclosure form at www.icmje.org/coi_disclosure.pdf. There are no relevant conflicts of interest, financial or other types of relationships that may influence the manuscript declared by authors. Authors do not have any patents and are not associated to any conditions or circumstances that may lead to conflicts of interest.

Funding statement

This work was funded by EIT Health (Grant 18654).

References

For peer review only

1. HealthIT.gov. Basics of Health IT. https://www.healthit.gov/patients-families/basics-health-it Accessed Feb 6 2018.
2. American College of Preventive Medicine. Preventive Medicine [Internet]. acpm.org. [cited 2018 Mar 27]. Available from: http://www.acpm.org/page/preventivemedicine
3. Kickbusch I, Nutbeam D. Health promotion glossary. World Health Organization; 1998.
4. Vodopivec-Jamsek V, de Jongh T, Gurol-Urganci I, Atun R, Car J. Mobile phone messaging for preventive health care. Cochrane Database Syst Rev. 2012;doi:10.1002/14651858.CD007457.pub2
5. Gurol-Urganci I, de Jongh T, Vodopivec-Jamsek V, Car J, Atun R. Mobile phone messaging for communicating results of medical investigations. Cochrane Database Syst Rev. 2012;doi:10.1002/14651858.CD007456.pub2
6. Whittaker R, McRobbie H, Bullen C, Rodgers A, Gu Y. Mobile phone-based interventions for smoking cessation. Cochrane Database Syst Rev. 2016; doi:10.1002/14651858.CD006611.pub4
7. Chaudhry B, Wang J, Wu S, Maglione M, Mojica W, Roth E, et al. Systematic review: impact of health information technology on quality, efficiency, and costs of medical care. Ann Intern Med. 2006;144(10):742–52.
8. Miriovsky BJ, Shulman LN, Abernethy AP. Importance of health information technology, electronic health records, and continuously aggregating data to comparative effectiveness research and learning health care. J Clin Oncol Off J Am Soc Clin Oncol. 2012;doi:10.1200/JCO.2012.42.8011
9. Rodgers A, Corbett T, Bramley D, Riddell T, Wills M, Lin R-B, et al. Do u smoke after txt? Results of a randomised trial of smoking cessation using mobile phone text messaging. Tob Control. 2005;doi: 10.1136/tc.2005.011577
10. Buntin MB, Burke MF, Hoaglin MC, Blumenthal D. The Benefits Of Health Information Technology: A Review Of The Recent Literature Shows Predominantly Positive Results. Health Aff (Millwood). 2011;doi:10.1377/hlthaff.2011.0178
11. Posadzki P, Mastellos N, Ryan R, Gunn LH, Felix LM, Pappas Y, et al. Automated telephone communication systems for preventive healthcare and management of long-term conditions. Cochrane Database Syst Rev. 2016;doi:10.1002/14651858.CD009921.pub2
12. Cocosila M, Archer N, Haynes RB, Yuan Y. Can wireless text messaging improve adherence to preventive activities? Results of a randomised controlled trial. Int J Med Inf. 2009;doi:10.1016/j.ijmedinf.2008.07.011
13. Shapiro JR, Bauer S, Hamer RM, Kordy H, Ward D, Bulik CM. Use of text messaging for monitoring sugar-sweetened beverages, physical activity, and screen time in children: a pilot study. J Nutr Educ Behav. 2008;doi:10.1016/j.jneb.2007.09.014
14. Buller DB, Borland R, Woodall WG, Hall JR, Hines JM, Burris-Woodall P, et al. Randomized trials on consider this, a tailored, internet-delivered smoking prevention program for

For peer review only

adolescents. Health Educ Behav Off Publ Soc Public Health Educ. 2008;doi:10.1177/1090198106288982

15. Kasatpibal N, Viseskul N, Srikantha W, Fongkaew W, Surapagdee N, Grimes RM. Effects of Internet-based instruction on HIV-prevention knowledge and practices among men who have sex with men. Nurs Health Sci. 2014;doi:10.1111/nhs.12135
16. Rerksuppaphol L, Rerksuppaphol S. Internet Based Obesity Prevention Program for Thai School Children- A Randomized Control Trial. J Clin Diagn Res JCDR. 2017;doi:10.7860/JCDR/2017/21423.9368
17. Lewis BA, Gjerdingen DK, Avery MD, Guo H, Sirard JR, Bonikowske AR, et al. Examination of a telephone-based exercise intervention for the prevention of postpartum depression: design, methodology, and baseline data from The Healthy Mom study. Contemp Clin Trials. 2012;doi:10.1016/j.cct.2012.07.015
18. Fukuoka Y, Gay CL, Joiner KL, Vittinghoff E. A Novel Diabetes Prevention Intervention Using a Mobile App: A Randomized Controlled Trial With Overweight Adults at Risk. Am J Prev Med. 2015;doi:10.1016/j.amepre.2015.01.003
19. Arksey H, O'Malley L. Scoping studies: towards a methodological framework. Int J Soc Res Methodol. 2005;doi:10.1080/1364557032000119616

For peer review only

For peer review only - http://bmjopen.bmj.com/site/about/guidelines.xhtml

Appendices:

Appendix 1: Table displaying the PRISMA-P 2015 Checklist

PRISMA-P 2015 Checklist

This checklist to be used for the Systematic Reviews protocol submission was adapted from Table 3 in Moher D et al: Preferred reporting items for systematic review and meta-analysis protocols (PRISMA-P) 2015 statement. Systematic Reviews 2015 4:1

Section and Topic	#	Checklist Item	Information reported		Page number(s)
			Yes	No	
**Administrative information**					
**Title**					
identification	1a	Identify the report as a protocol of a systematic review	☐	☒	
Update	1b	If the protocol is for an update of a previous systematic review, identify as such	☐	☒	
**Registration**	2	If registered, provide the name of the registry (such as PROSPERO) and registration number	☐	☒	
**Authors**					
Contact	3a	Provide name, institutional affiliation, e-mail address of all protocol authors; provide physical mailing address of corresponding author	☒	☐	1
Contributions	3b	Describe contributions of protocol authors and identify the guarantor of the review	☒	☐	11
**Amendments**	4	If the protocol represents an amendment of a previously completed or published protocol, identify as such and list changes; otherwise, state plan for documenting important protocol amendments	☐	☒	
**Support**					
Sources	5a	Indicate sources of financial or other support for the review	☒	☐	11

peer review only

Section and Topic	#	Checklist Item	Information reported		Page number(s)
			Yes	No	
Sponsor	5b	Provide name for the review funder and/or sponsor	☒	☐	11
Role of Sponsor or Funder	5c	Describe roles of funder(s), sponsor(s), and/or institution(s), if any, in developing the protocol	☒	☐	11
**Introduction**					
Rationale	6	Describe the rationale for the review in the context of what is already known	☒	☐	3-4
Objectives	7	Provide an explicit statement of the question(s) the review will address with reference to participants, interventions, comparators, and outcomes (PICO)	☒	☐	6
**Methods**					
Eligibility criteria	8	Specify the study characteristics (such as PICO, study design, setting, time frame) and report characteristics (such as years considered, language, publication status) to be used as criteria for eligibility for the review	☒	☐	7-8
Information sources	9	Describe all intended information sources (such as electronic databases, contact with study authors, trial registers or other grey literature sources) with planned dates of coverage	☒	☐	6-7
Search strategy	10	Present draft of search strategy to be used for at least one electronic database, including planned limits, such that it could be repeated	☒	☐	17
Study records:					
Data management	11a	Describe the mechanism(s) that will be used to manage records and data throughout the review	☒	☐	7
Selection process	11b	State the process that will be used for selecting studies (such as two independent reviewers) through each phase of the review (that is, screening, eligibility and inclusion in meta-analysis)	☒	☐	7

Section and Topic	#	Checklist Item	Information	Page

For peer review only

For peer review only - http://bmjopen.bmj.com/site/about/guidelines.xhtml

			reported		number(s)
			Yes	No	
Data collection process	11c	Describe planned method of extracting data from reports (such as piloting forms, done independently, in duplicate), any processes for obtaining and confirming data from investigators	☒	☐	7, 18
**Data items**	12	List and define all variables for which data will be sought (such as PICO items, funding sources), any pre-planned data assumptions and simplifications	☒	☐	7-8
**Outcomes and prioritization**	13	List and define all outcomes for which data will be sought, including prioritization of main and additional outcomes, with rationale	☒	☐	7-10 18
**Risk of bias in individual studies**	14	Describe anticipated methods for assessing risk of bias of individual studies, including whether this will be done at the outcome or study level, or both; state how this information will be used in data synthesis	☐	☒	
**Data synthesis**	15a	Describe criteria under which study data will be quantitatively synthesised	☐	☒	
	15b	If data are appropriate for quantitative synthesis, describe planned summary measures, methods of handling data and methods of combining data from studies, including any planned exploration of consistency (such as I2 , Kendall's τ)	☐	☒	
	15c	Describe any proposed additional analyses (such as sensitivity or subgroup analyses, meta-regression)	☐	☒	
	15d	If quantitative synthesis is not appropriate, describe the type of summary planned	☐	☒	
**Meta bias(es)**	16	Specify any planned assessment of meta-bias(es) (such as publication bias across studies, selective reporting within studies)	☐	☒	
**Confidence in cumulative evidence**	17	Describe how the strength of the body of evidence will be assessed (such as GRADE)	☐	☒	

For peer review only

For peer review only - http://bmjopen.bmj.com/site/about/guidelines.xhtml

Appendix 2: Proposed MEDLINE Literature Search Strategy

**Concept**	**Medical Subject Headings (MeSH)**	**Search terms**
**Health Information Technologies**	Medical Informatics/	electronic patient record* OR electronic medical record* OR personal health record* OR Health information exchange or technology OR telemedicine OR text message* OR sms OR telephone OR computerized decision support system OR public health informatic* OR cellular phone* OR smartphone* OR mobile* OR ipad* or computer-assisted OR user-computer interface OR personal digital assistant OR computer* OR handheld OR electronic wearable device* OR electronic wearable technology OR data
**Primary Prevention**	Quality of Life/<br>tobacco use/<br>smoking/<br>dietary services/<br>preventive health services/ early intervention (education)/<br>early medical intervention/<br>health education/<br>primary prevention/<br>immunization/	exercise OR physical activity OR diet OR healthy behavior* OR weightloss OR weight change OR weight reduction OR weight management OR weight gain OR smoking cessation OR disease prevention

For peer review only

Appendix 3: Data abstraction form

**Reviewer**			**Date**
**Scoping review of Health information technology used for primary prevention in preventive medicine**			
**Publication Information**			
**Study**		**First Author**	
**Year of Publication**	**Journal**		
**Country**	**Discipline**		
**Health information technology(ies) studied**			
**Objective 1**	**General description of the health information technology(ies) studied**		
	**The primary prevention purpose of the health information technology**		
**Objective 2**	**Primary prevention patient outcome(s) studied**		
**Objective 3**	**Is there a comparator to the health information technology, if so, how is it different than the comparator?**		

For peer review only

For peer review only - http://bmjopen.bmj.com/site/about/guidelines.xhtml
